# Preventive Effects of Lidocaine, Fentanyl, Acetaminophen, and TENS on Propofol-Induced Injection Pain: A Randomized Double-Blind Clinical Trial

**DOI:** 10.5812/aapm-165776

**Published:** 2025-10-14

**Authors:** Behzad Nazemroaya, Mehran Rezvani, Mohaddeseh Goli, Mozhdeh Salimipoor

**Affiliations:** 1 Department of Anesthesiology and Critical Care, Isfahan University of Medical Sciences, Isfahan, Iran; 2 School of Medicine, Isfahan University of Medical Sciences, Isfahan, Iran

**Keywords:** Transcutaneous Electrical Nerve Stimulation, Pain, Preventive, Lidocaine, Fentanyl, Acetaminophen, Propofol

## Abstract

**Background:**

Propofol is one of the most commonly used drugs in anesthesia, but administering it to patients often causes significant pain and discomfort. Numerous studies have been conducted on various methods to mitigate this adverse effect due to its prevalence.

**Methods:**

This study was conducted in 2025 at Kashani Hospital in Isfahan, Iran, involving 150 patients undergoing elective surgery who received propofol for anesthesia induction. They were randomly assigned to five groups: Normal saline (control), transcutaneous electrical nerve stimulation (TENS), acetaminophen, intravenous lidocaine, or intravenous fentanyl. Additionally, this randomized, double-blind, controlled clinical trial (allocation ratio 1:1) employed block randomization. Patients, along with the assessor and the data analyst, were blinded. Statistical analyses were performed using ANOVA, chi-square, and repeated-measures ANOVA. Pain during injection was rated using a 0 - 10 numerical scale.

**Results:**

There was no statistically significant difference in pain intensity between the intervention groups when comparing their mean pain scores (P > 0.05). However, in most comparisons, the normal saline group had the highest average pain scores.

**Conclusions:**

All interventions except saline reduced propofol injection pain. Secondary outcomes, including patient satisfaction, recovery time, and hemodynamic stability, were also evaluated, and no significant adverse effects were observed among the interventions. These findings support the use of multiple safe strategies to minimize discomfort during anesthesia induction.

## 1. Background

Propofol is one of the most commonly used drugs for the induction and maintenance of anesthesia. It is popular due to its rapid onset and quick recovery. However, more than 70% of patients experience pain during the injection, which is the seventh most distressing anesthesia-related experience and the third most common problem in outpatient surgery (1, 2). This pain can trigger the body’s stress response, potentially interfering with anesthesia and putting the patient's safety at risk.

The exact mechanism is not well understood, but the drug's aqueous phase may interact with the endothelium and release peptides (3). Methods to decrease this pain include altering the speed of the injection, using carrier fluids (4), changing the solvents (5), and administering analgesics concurrently. Lidocaine, tramadol, ondansetron, alfentanil, remifentanil, and their combinations with propofol (6, 7) are some of the most common agents used. The most common method is lidocaine pretreatment (1), but acetaminophen has also been shown to be effective, possibly through central and peripheral mechanisms (8, 9). A study from 2022 found that administering acetaminophen and lidocaine intravenously was more effective than administering lidocaine alone (10).

Transcutaneous electrical nerve stimulation (TENS) is a non-invasive method that has been used to alleviate pain from propofol injections, especially when combined with lidocaine. It may also aid in recovery after surgery (2, 11). Researchers have also investigated fentanyl, a short-acting opioid agonist that acts on both the central and peripheral nervous systems to relieve pain (12, 13).

## 2. Objectives

This study aimed to compare the effects of pretreatment with lidocaine, intravenous acetaminophen, intravenous fentanyl, and TENS on pain reduction during propofol injection. This comparison was necessary because propofol injection pain is very common, and minimizing anesthesia-related discomfort is essential.

## 3. Methods

The Ethics Committee of Isfahan University of Medical Sciences (IR.MUI.MED.REC.1403.052) and the Iranian Registry of Clinical Trials (IRCT20160307026950N64) both approved this study, which was a randomized, double-blind, controlled clinical trial with a parallel control group. All patients provided written informed consent. The allocation ratio between the study groups was 1:1, and randomization was performed using a block randomization method via www.random.org. The study took place in 2025 at Kashani Hospital in Isfahan, Iran. A Numerical Rating Scale (NRS) was used to measure pain from propofol injection, where a score of 0 indicated "no pain", and a score of 10 indicated "the worst pain you can imagine".

Inclusion criteria were all patients between the ages of 18 and 65 who were admitted for elective surgery and received propofol for anesthesia induction. Exclusion criteria included renal or hepatic disease, confusion, dementia, inability to speak, acute or chronic pain, a history of allergy to any study drug, and pregnancy.

Withdrawal criteria included a change in anesthesia technique, inability to cannulate the correct vein, and the patient not responding appropriately after receiving the drug. We used a convenience sampling method to select samples. Using the sequence generator option in the Numbers section of the website www.random.org, we randomly assigned numbers 1 - 150 into five groups. This is how the study groups were chosen for all eligible patients. The study was conducted in a double-blind manner, meaning that the patient, the statistical consultant, and the evaluator were unaware of the group assignments. There were five groups in the study:

- Control group: Two mL of normal saline was injected.

- Lidocaine group: Two percent lidocaine at a dose of 0.5 mg/kg.

- Fentanyl group: 1.5 μg/kg of fentanyl (50 μg/mL).

- Acetaminophen group: 150 mg/mL (10 mg/kg) acetaminophen.

- The TENS group: TENS.

To ensure the infusion volume was similar for everyone, the five groups received their assigned intervention as follows: The control group received 100 mL of normal saline; the lidocaine group received 0.025 mL/kg of lidocaine, plus enough normal saline to make a total of 100 mL; the fentanyl group received 0.03 mL/kg of fentanyl, plus normal saline to make 100 mL; the acetaminophen group received 0.07 mL/kg of acetaminophen, with normal saline added to make a total of 100 mL; the TENS group received 100 mL of normal saline. All patients were prepared for surgery according to standard procedures. An individual not involved in the study applied transcutaneous electrical stimulation to the TENS group 10 minutes before administering propofol 1% (Fresenius Kabi, Germany). We used a TENS device (EM 80 model, Beurer) with 45 × 45 mm adhesive electrodes. The electrodes were placed 5 cm beyond the site of intravenous cannulation, in the cubital region. All patients had two electrodes applied. The TENS group had the device turned on, while the other groups had it turned off. To maintain the blinding effect, the device was covered after it was turned on or off. The TENS settings were a pulse width of 200 μs and a frequency of 100 Hz, with intensity adjusted to produce a tingling sensation without pain, as reported by the patient.

During patient preparation and before the study began, the medical team placed an 18G cannula in the right cubital vein of all groups. The 100 mL solution (normal saline, acetaminophen, fentanyl, or lidocaine, depending on the group) was administered over a period of 10 minutes. Thereafter, a dose of 2.5 mg/kg of 1% propofol was given. Patients were asked to rate the severity of their pain on the NRS (0 - 10) every 5 seconds after the propofol injection started until they were completely unconscious. We recorded the following information for each patient: Demographic information (sex, age, height, weight), pain scores reported by the patient, the type and length of surgery, the time it took to recover, and the patient's heart rate (HR), mean arterial pressure (MAP), and oxygen saturation (SpO_2_) before and after anesthesia induction. Blinding was performed such that the patient, the outcome assessor, and the data analyst were all unaware of the assigned intervention group.

### 3.1. Statistical Analysis

We used SPSS Statistics version 23.0 (IBM Corp., Armonk, NY, USA). The chi-square test or Fisher's exact test was used to compare categorical variables between groups when appropriate. The Shapiro-Wilk test was employed to verify if continuous variables were normally distributed. Mean ± standard deviation (SD) was used to present normally distributed data, and median [interquartile range (IQR)] was used for non-normally distributed data. For the main outcome (pain scores taken at different times during propofol injection), we used repeated measures analysis of variance (RM-ANOVA) for normally distributed data and the Friedman test for non-normally distributed data. The Greenhouse-Geisser correction was applied when the sphericity assumption in RM-ANOVA was not met. Post-hoc pairwise comparisons between groups were performed using the Bonferroni adjustment for normally distributed data and Dunn’s test for non-normally distributed data. We used one-way ANOVA for normally distributed data and the Kruskal-Wallis test for non-normally distributed data to compare baseline continuous variables like age and BMI. RM-ANOVA was used for normally distributed data and the Friedman test for non-normally distributed data to examine changes in hemodynamic parameters (HR and MAP) over time. A P-value of less than 0.05 was considered statistically significant.

## 4. Results

**Figure 1.**
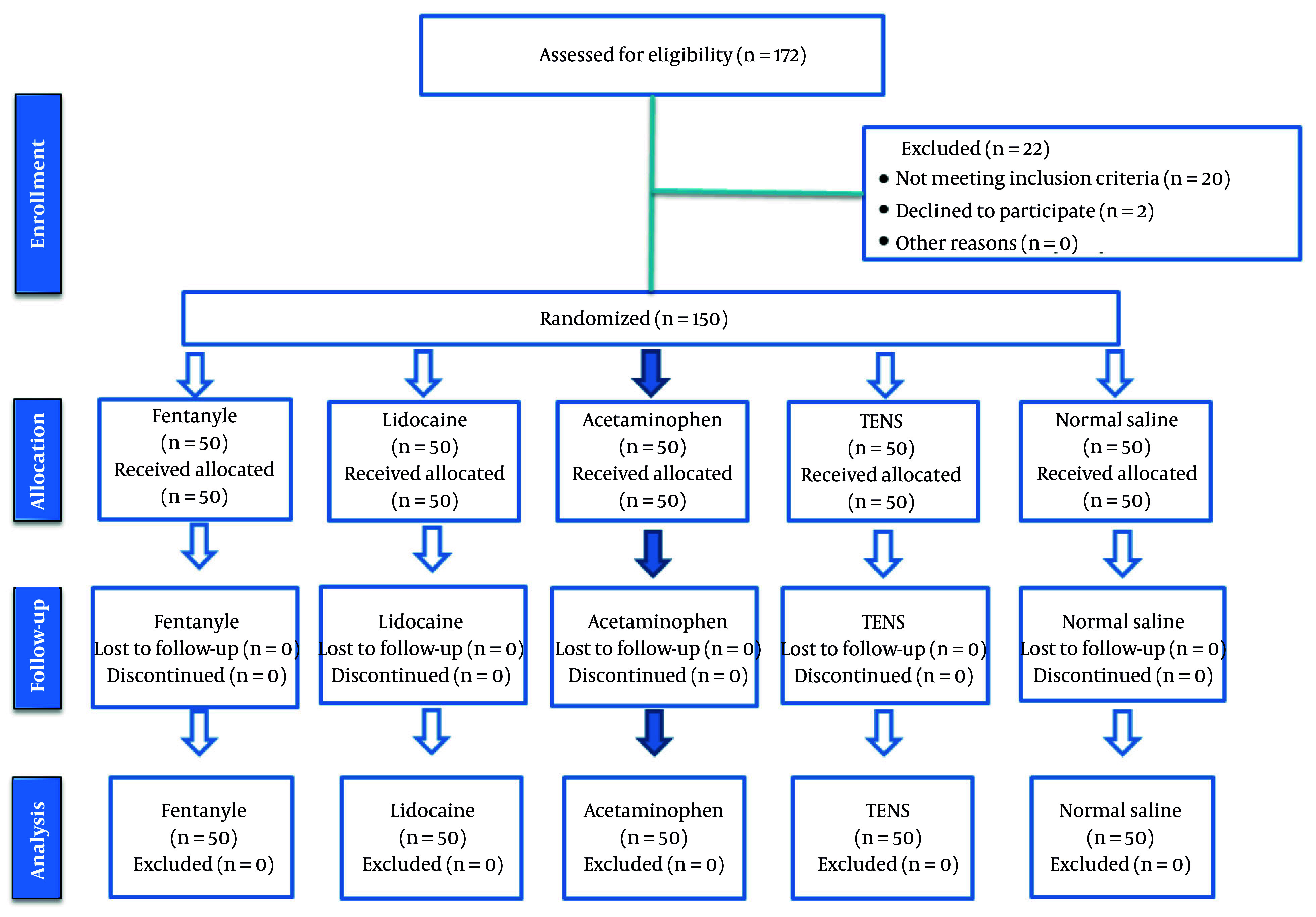
Flow chart

This study included 150 patients who were candidates for elective surgeries, with thirty individuals randomly assigned to each of the five groups (Figure 1). 

The average age of the patients ranged from 35 to 39 years, and their average BMI ranged from 24.7 to 26.9. The Kruskal-Wallis test showed no statistically significant difference between the intervention groups in terms of age (P = 0.935) or BMI (P = 0.766). The lidocaine group had the highest percentage of women (43.3%), while the TENS group had the lowest (10%). Overall, 30.7% of the participants were women, and 69.3% were men. There was no statistically significant difference (P = 0.273) in the chi-square analysis of sex distribution among the groups (Table 1). 

**Table 1.** Basic and Clinical Characteristics of Patients in the Five Groups ^a^

Variables	Fentanyl	Lidocaine	Acetaminophen	TENS	Normal Saline	P-Value
**Sex; 150 (100)**						0.273
Male; 105 (70)	24 (22.8)	11 (10.4)	23 (22)	25 (23.8)	22 (21)	
Female; 45 (30)	6 (13.3)	19 (42.2)	7 (15.5)	5 (11.1)	8 (17.7)	
**Age (y)**	37.44 ± 9.75	37.73 ± 10.77	35.07 ± 10.18	36.07 ± 10.18	39.07 ± 10.18	0.960
**Weight (kg)**	68.70 ± 7.60	66.62 ± 11.35	75.31 ± 8.11	71.21 ± 9.32	69.70 ± 7.60	0.525
**BMI**	24.70 ± 7.60	23.30 ± 5.41	25.67 ± 8.89	24.67 ± 8.89	26.9 ± 5.11	0.935

Abbreviation: TENS, transcutaneous electrical nerve stimulation.

^a^ Values are expressed as No. (%) or mean ± standard deviation (SD).

**Figure 2.**
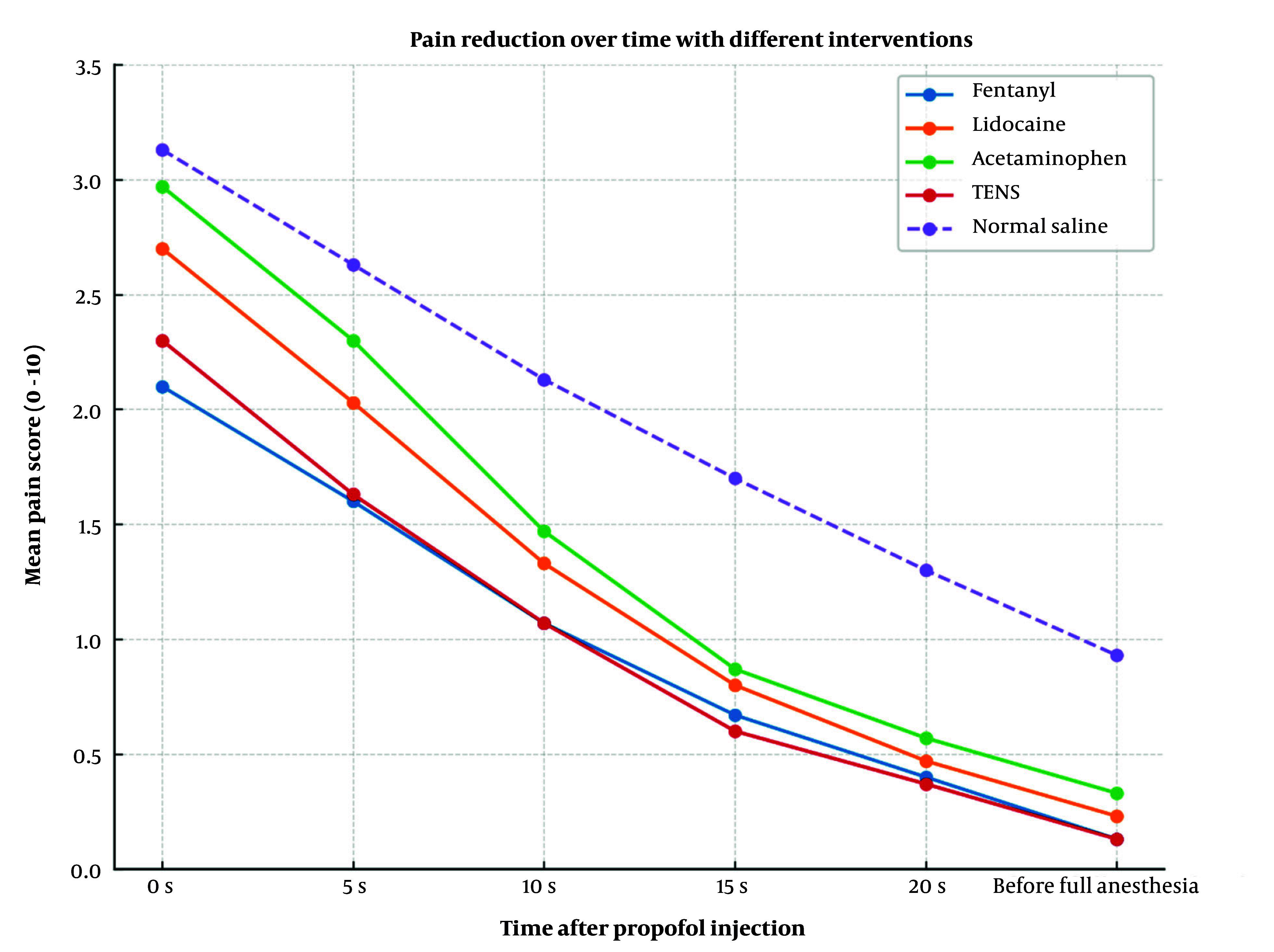
Mean pain scores over time after propofol injection in patients receiving fentanyl, lidocaine, acetaminophen, transcutaneous electrical nerve stimulation (TENS), or normal saline

This confirms that the groups being compared were similar in terms of their baseline demographic characteristics, which enhances the validity of the comparisons of intervention effects. The results of the within-subject analysis of variance indicated that all patients' pain levels significantly decreased over time (P < 0.001), regardless of the treatment type. The time × group interaction was also statistically significant (P = 0.005), indicating that the groups exhibited different patterns of pain relief (Figure 2). 

This means that each intervention had a different effect on the reduction of pain levels following the propofol injection. These results indicate that both the time and the type of treatment influenced the effectiveness of injection pain control.

The P-value was never less than 0.05 in any of the pairwise comparisons, so the differences in mean pain intensity between groups were not statistically significant. The normal saline group had the largest negative differences in most comparisons (indicating higher pain scores), but these differences were not statistically significant (Table 2). 

**Table 2.** Mean Pain Intensity Before Injection and then Every 5 Seconds Up to 10 Seconds After Completion of Propofol Injection Until Full Anesthesia in Each of the Five Groups ^a^

Variables; Time	Fentanyl	Lidocaine	Acetamin	TENS	Normal Saline
**Pain**					
Start of injection	2.10 ± 2.24	2.70 ± 2.13	2.97 ± 2.03	2.30 ± 1.97	3.13 ± 2.34
5th injection	1.60 ± 1.94	2.03 ± 1.96	2.30 ± 1.92	1.63 ± 1.69	2.63 ± 2.16
10th injection	1.07 ± 1.79	1.33 ± 1.82	1.47 ± 1.72	1.07 ± 1.36	2.13 ± 2.00
15th injection	0.67 ± 1.58	0.80 ± 1.65	0.87 ± 1.55	0.60 ± 1.18	1.70 ± 1.91
20th injection	0.40 ± 1.35	0.47 ± 1.38	0.57 ± 1.43	0.37 ± 0.95	1.30 ± 1.84
Before induction	0.13 ± 0.56	0.23 ± 0.80	0.33 ± 0.90	0.13 ± 0.52	0.93 ± 1.77
**P-value**					
Between subjects	0.001 ^b^	-	-	-	-
Within subjects	0.248 ^c^	-	-	-	-
Time × group	0.005 ^d^	-	-	-	-

Abbreviation: TENS, transcutaneous electrical nerve stimulation.

^a^ Values are expressed as mean ± standard deviation (SD).

^b^ P-value between-group indicates the overall difference in mean values among the independent groups (group effect).

^c^ P-value within-group indicates the significance of changes over time within each group (time effect).

^d^ P-value time × group indicates whether the pattern of changes over time differs between groups (interaction effect).

Before and after the propofol injection, the average HR of patients in all five groups changed slightly. In some groups, the HR decreased slightly after the injection. However, according to statistical analysis, these changes were not significant (P = 0.073 for the time effect). Furthermore, the comparisons between groups and the time × group interaction were not significant (P > 0.05), indicating that the type of intervention did not have a significant effect on HR changes.

When examining MAP before and after propofol injection, within-subject analysis showed a significant effect of time (P < 0.001), indicating that MAP decreased after the intervention compared to baseline. There was also a significant time × group interaction (P = 0.009), suggesting that the amount of MAP change differed between the treatment groups. However, when examining the differences between the groups without considering time, there was no statistically significant difference (P = 0.188). The results show that injecting propofol significantly reduced blood pressure, and the type of intervention influenced the extent of this change (Table 3). 

**Table 3.** Comparison of Mean Hemodynamic Parameters of Patients Between Five Groups (N = 30) ^a^

Variables	Fentanyl	Lidocaine	Acetaminoph	TENS	Control	P-Value
**HR**						
Start of injection	96.02 ± 11.88	98.53 ± 10.56	94.22 ± 7.23	98.37 ± 6.68	98.37 ± 6.68	0.575
5th s injection	98.37 ± 6.68	98.28 ± 12.25	97.51 ± 15.58	98.37 ± 6.68	98.37 ± 6.68	0.863
10th s injection	98.37 ± 6.68	98.57 ± 8.46	94.60 ± 15.34	99.42 ± 6.13	99.42 ± 6.13	0.973
15th s injection	99.42 ± 6.13	98.82 ± 8.68	98.48 ± 17.83	100.0 ± 4.65	100.0 ± 4.65	0.371
20th s injection	100.0 ± 4.65	99.88 ± 9.85	100.97 ± 16.28	100.4 ± 4.67	100.4 ± 4.67	0.255
Before induction	100.4 ± 4.67	90.04 ± 9.88	94.19 ± 11.22	98.47 ± 6.78	98.47 ± 6.78	0.530
P-value	0.023	0.90	0.61	0.23	0.70	0.073
**Mean blood pressure**						
Start of injection	100.42 ± 14.64	99.06 ± 9.21	101.71 ± 8.85	98.71 ± 87.13	99.14 ± 5.28	0.550
5th s injection	85.11 ± 12.59	84.71 ± 87.13	87.13 ± 12.33	82.73 ± 8.29	85.45 ± 6.72	0.591
10th s injection	83.88 ± 11.79	82.73 ± 8.29	84.92 ± 6.01	82.93 ± 7.98	80.71 ± 8.85	0.186
15th s injection	83.6 ± 9.12	82.93 ± 7.98	83.85 ± 6.85	81.9 ± 6.97	87.13 ± 12.33	0.599
20th s injection	84.35 ± 9.58	81.9 ± 6.97	84.14 ± 5.28	85.42 ± 14.64	84.92 ± 6.01	0.386
Before induction	82.85 ± 9.8	80.51 ± 5.95	85.45 ± 6.72	85.11 ± 12.59	83.85 ± 6.85	0.770
P-value	0.09	0.017	0.013	0.017	0.019	0.235
**SPO**_**2**_						
Start of injection	98.92 ± 1.02	98.23 ± 2.60	98.19 ± 2.40	98.54 ± 1.07	99.04 ± 0.66	0.179
5th s injection	98.92 ± 1.02	98.50 ± 1.79	98.50 ± 1.61	98.65 ± 0.94	99.08 ± 0.63	0.560
10th s injection	99.04 ± 0.66	98.50 ± 1.82	98.65 ± 1.65	98.96 ± 0.60	99.27 ± 0.60	0.411
15th s injection	99.08 ± 0.63	98.68 ± 1.36	98.77 ± 1.31	99.00 ± 0.63	99.23 ± 0.59	0.274
20th s injection	99.27 ± 0.60	98.86 ± 0.99	98.92 ± 0.98	99.04 ± 0.53	98.92 ± 0.98	0.726
Before induction	99.23 ± 0.59	99.05 ± 0.65	99 ± 0.69	99.08 ± 0.63	99 ± 0.69	0.506
P-value	0.012	0.008	0.022	< 0.001	0.006	0.040
**Systolic blood pressure**						
Start of injection	132.63 ± 18.53	127.30 ± 15.04	128.20 ± 18.09	127.50 ± 16.48	127.30 ± 15.04	0.585
5th s injection	123.70 ± 30.26	126.10 ± 15.75	127.77 ± 29.22	127.00 ± 18.25	126.10 ± 15.75	0.924
10th s injection	124.67 ± 33.04	123.73 ± 27.62	137.30 ± 25.75	124.10 ± 23.27	116.00 ± 18.69	0.171
15th s injection	116.00 ± 18.69	117.63 ± 16.53	112.80 ± 20.69	115.90 ± 16.56	117.03 ± 16.86	0.776
20th s injection	117.03 ± 16.86	116.73 ± 19.42	119.03 ± 18.09	114.77 ± 16.57	123.70 ± 30.26	0.833
Before induction	117.90 ± 16.22	114.04 ± 18.11	119.70 ± 14.67	117.60 ± 17.60	124.67 ± 33.04	0.638
P-value	0.005	0.006	< 0.001	0.006	0.040	0.003
**Diastolic blood pressure**						
Start of injection	77.88 ± 10.23	82.70 ± 12.24	79.56 ± 11.29	77.32 ± 11.78	71.93 ± 10.88	0.275
5th s injection	74.62 ± 14.98	80.57 ± 12.55	77.11 ± 12.56	76.61 ± 12.84	70.85 ± 11.80	0.463
10th s injection	77.31 ± 19.69	82.39 ± 13.50	82.11 ± 21.65	79.25 ± 14.29	82.70 ± 12.24	0.090
15th s injection	67.73 ± 14.95	76.09 ± 12.06	68.81 ± 11.24	71.82 ± 9.88	80.57 ± 12.55	0.713
20th s injection	68.69 ± 16.21	68.43 ± 14.24	71.93 ± 10.88	71.61 ± 11	82.39 ± 13.50	0.153
Before induction	68.15 ± 13.33	66.35 ± 14.41	70.85 ± 11.80	70.57 ± 17.76	82.11 ± 21.65	0.075
P-value	< 0.001	< 0.001	< 0.001	< 0.001	< 0.001	< 0.001

Abbreviations: TENS, transcutaneous electrical nerve stimulation; HR, heart rate; SpO_2_, oxygen saturation.

^a^ Values are expressed as No. (%) or mean ± standard deviation (SD).

Fifteen patients (10%) experienced apnea after receiving propofol, with the TENS group having the most cases (33.3%). The chi-square test indicated that the only statistically significant difference between the groups was the number of apneas (P < 0.001). There were no statistically significant differences between treatment groups for other adverse effects, such as headache (P = 0.488), thrombophlebitis (P = 0.388), and allergy (P = 0.515). These results suggest that individuals who used TENS had a significantly higher rate of apnea, while other undesirable effects were more evenly distributed among the groups (Table 4). 

**Table 4.** Determining the Frequency of Side Effects in Five Group ^a^

Sid Effects Group	Fentanyl (N = 30)	Lidocaine (N = 30)	Acetaminoph (N = 30)	TENS (N = 30)	N/S (N = 30)	Total (N = 150)	P-Value
**Headache**	2 (6.7)	3 (10)	0 (0)	1 (3.3)	2 (6.7)	8 (5.3)	0.488
**Apnea**	10 (33.3)	3 (10)	1 (3.3)	1 (3.3)	0 (0)	15 (10)	< 0.001
**Thrombophlebitis**	2 (6.7)	1 (3.3)	2 (6.7)	0 (0)	0 (0)	5 (3.3)	3.888
**Allergy**	4 (13.3)	3 (10)	1 (3.3)	1 (3.3)	3 (10)	12 (8)	0.515

Abbreviation: TENS, transcutaneous electrical nerve stimulation.

^a^ Values are expressed as No. (%).

Because our study did not detect any comparisons with a P-value below 0.05, the differences in mean pain intensity between groups were not statistically significant. Although the normal saline group had the largest negative difference in most comparisons (indicating higher pain scores), all of the intervention groups were more effective at reducing pain than normal saline. However, there were no significant differences between the groups that received the interventions. Therefore, when attempting to make propofol injections less painful, we should consider the potential adverse effects of these treatments when selecting the most appropriate one.

## 5. Discussion

Valizadeh et al.'s study surveyed three methods to compare the severity of pain from propofol injection: Mixing propofol with lidocaine, administering propofol after lidocaine, and injecting pure 1% propofol. The results indicated that pain was less severe when propofol was administered after lidocaine than when it was mixed with lidocaine. They concluded that administering 40 mg of 2% lidocaine before the procedure was more effective than mixing the same amount with propofol (14). Our study indicated that lidocaine was significantly more effective than normal saline at reducing pain from propofol injections, which is consistent with these results.

Similarly, Eydi et al. studied the effects of lidocaine and nitroglycerin together and lidocaine alone on patients who received general anesthesia. The hemodynamic variables and arterial SpO_2_ remained stable in all three groups. Both lidocaine alone and the lidocaine-nitroglycerin combination reduced the incidence of propofol injection pain. The combination was more effective and caused less pain (15). Our results are in agreement, further supporting the evidence that lidocaine is more effective at relieving pain than a placebo.

Notably, some randomized trials have reported that alternative agents such as nalbuphine can reduce propofol injection pain compared with lidocaine (16), highlighting that agent choice and dosing strategy may materially influence outcomes across settings. Our pilot did not evaluate nalbuphine; therefore, the external validity of our findings should be interpreted alongside such evidence and verified in adequately powered studies.

The study by Kolahdouzan et al. examined remifentanil for preventing pain from intravenous propofol injections and found that it was as effective as lidocaine. This suggests that remifentanil could be used instead of lidocaine if an opioid is already part of the anesthetic plan (17). Conversely, our results showed that lidocaine and fentanyl did not significantly differ in their effectiveness at reducing induction pain from propofol.

Jin et al. studied TENS and lidocaine together to alleviate pain from propofol injections in 220 women undergoing hysteroscopic surgery. Their results indicated that TENS and lidocaine together significantly reduced both the incidence and severity of pain more than lidocaine alone (2). Our study found that TENS can help with propofol injection pain, even when used alone.

Sedighinejad et al. examined 220 orthopedic surgery patients and compared alfentanil, magnesium sulfate, and ketamine to determine which was most effective for alleviating pain during intravenous propofol injection. After accounting for demographic factors, there were no significant differences in pain severity between the groups (18). This aligns with our findings: There were no statistically significant differences between the different interventions tested, even though all active treatments tended to lower pain scores compared to saline. Recent evidence has also highlighted the analgesic potential of lidocaine patch formulations in perioperative settings (19).

Overall, these results support the idea that no single method was clearly superior to the others in our trial for reducing propofol injection pain. This includes lidocaine, opioids, paracetamol, and TENS. Variations in patient demographics, dosing schedules, and drug administration methods may explain why the results of different studies are not always consistent.

### 5.1. Clinical Implications

Since propofol is frequently used for induction, methods to reduce injection pain remain important in clinical practice. In our study, all treatments except saline reduced pain. This finding suggests that the choice of method should depend on the patient's other health conditions, potential side effects, and the availability of the drugs. The TENS and other non-drug options may be especially beneficial for individuals who prefer not to take medications before surgery.

## Data Availability

The dataset presented in the study is available on request from the corresponding author during submission or after publication. The data are not publicly available due to the privacy.

## Clinical Trial Registration Code:

IRCT20160307026950N64.
